# COVID-19 in patients with severe mental illness: An analysis of in-patients at a psychiatric hospital in Cape Town

**DOI:** 10.4102/sajpsychiatry.v31i0.2286

**Published:** 2025-01-15

**Authors:** Haseena B. Sablay, Qhama Z. Cossie, Deirdre I. Pieterse

**Affiliations:** 1 Department of Psychiatry and Mental Health, Faculty of Health Sciences, University of Cape Town, Cape Town, South Africa

**Keywords:** COVID-19, outbreak, infectious disease, SARS-COV2, coronavirus, psychiatric hospital, severe mental illness

## Abstract

**Background:**

Psychiatric patients in specialist units are more vulnerable to infections such as SARS-COV-2 (severe acute respiratory syndrome coronavirus 2) because of hospital infrastructure and patients’ mental health.

**Aim:**

This study aimed to describe the psychiatric and medical profile, and the risk factors associated with more severe disease and clinical outcomes of coronavirus disease 2019 (COVID-19) in patients with severe mental illness (SMI) admitted to a specialist psychiatric hospital in South Africa between 01 April 2020 and 30 September 2021.

**Setting:**

The study was executed at the Vàlkenberg Hospital (VBH), which is a government-funded, specialised psychiatric hospital. The hospital comprises 370 beds made up of 145 forensic service beds and 225 acute service beds. It provides in-patient and out-patient services.

**Methods:**

Demographic and clinical information were collected for all VBH in-patients who tested positive for SARS-COV-2 from 01 April 2020 to 30 September 2021.

**Results:**

A total of 254 participants tested positive for SARS-COV-2. The sample comprised 75% (*n* = 191) males with a mean age of 35.7 years. Most patients were diagnosed with schizophrenia (37%, *n* = 94), bipolar disorder (21%, *n* = 54) and schizoaffective disorder (19%, *n* = 49). Reported comorbidities included nicotine use (71%, *n* = 181), hypertension (11%, *n* = 28) and human immunodeficiency virus (7%, *n* = 18). Most patients (62%, *n* = 156) were symptomatic for COVID-19. Seven per cent (*n* = 17) required transfer to a medical ward. Almost all patients (99%, *n* = 252) recovered and 1% (*n* = 2) died.

**Conclusion:**

Contrary to early fears of high mortality among institutionalised SMI patients, most experienced mild COVID-19 illness and recovered.

**Contribution:**

This descriptive study provided information on in-patients with COVID-19 disease at a specialised psychiatric hospital during the pandemic.

## Introduction

Outbreaks of infectious diseases are common in psychiatric units, often caused by agents prevalent in the surrounding community.^1,2^ Unfortunately, psychiatric units are often ill prepared to manage infectious outbreaks, and secondary spread is all too common, making containment challenging.^1,3,4^ At the start of the pandemic, mental health practitioners in Wuhan encountered difficulties because of lack of guidelines, resources and insufficient training to provide mental health services during the coronavirus disease 2019 (COVID-19) pandemic.^5^ Clinicians in psychiatric hospitals feared that institutionalised patients would be at greater risk for contracting COVID-19 and have poorer outcomes than people in the community.^6,7^

### Factors influencing increased COVID-19 risk in psychiatric hospitals

Psychiatric hospitals are designed differently from general hospitals.^8^ They focus on therapeutic activities, have limited medical services and do not prioritise infection control.^5,9,10,11,12,13^ High patient turnover and shared workspaces are common.^8,12^ The wards have poor ventilation and may be over capacity, increasing the risk of infection.^7,10,11^ Inadequate COVID-19 screening for patients and staff may further exacerbate the risk.^2^

Aside from this, patient factors also play a significant role in the increased risk of contracting infectious diseases. Lifestyle choices, diet, lack of exercise and smoking increase the risk of physical health issues in patients with mental illness.^5,9,14,15^ Psychosocial factors like poor self-care, isolation and frequent healthcare visits can also impact their health.^5,7,9,10^ Patients may struggle to express and monitor symptoms, leading to longer hospital stays and difficulty adhering to protective measures.^8,9,10,15^

Clinical staff in a psychiatric hospital may not adequately respond to an infectious outbreak because of the lack of necessary infrastructure, equipment and training to manage infectious illnesses.^8,11,15^ Additionally, their focus on treating psychiatric illnesses may lead to medical symptoms being missed.^8^ There were challenges with staff adhering to policies such as social distancing, particularly in recreational areas like tea rooms.^8^ Furthermore, staff often work when they are mildly unwell and possibly infectious, which increases the risk of outbreaks within the facility.^8^

### The effects of the COVID-19 pandemic on the severe mental illness population

The COVID-19 pandemic put the severe mental illness (SMI) population at risk of relapse because of poor adherence to treatment, less access to medication and loneliness caused by lockdowns and COVID-19 diagnosis.^10,14,16^ Evidence suggested increased cigarette and alcohol use in general during the COVID-19 pandemic.^17^ The added stress of increased childcare responsibilities, social isolation, loss of income and the death of loved ones worsened the long-term prognosis of SMI.^10,14^

South Africa, which already had limited mental health resources, faced even greater challenges during the COVID-19 pandemic.^10^ Inadequate funding, staff shortages and structural issues have long plagued the country’s mental health services.^10,18,19^ The pandemic intensified these problems, particularly for those with SMI.^10^

In-patient specialised psychiatric hospitals are key in supporting the SMI population by relieving symptoms and enhancing functioning in a physically and psychologically safe space.^10,16^ However, the COVID-19 pandemic disrupted these activities, leading to a significant impact on the delivery of care and services.^2,16,20^

### COVID-19 impact on in-patients in psychiatric units

Throughout the COVID-19 pandemic, policymakers overlooked the SMI population while concentrating globally on the psychological impact on the general population.^14,15^ The psychiatry service had to adapt to accommodate the need for continued service provision.^2,4,10^

During the pandemic’s early stages, psychiatric hospitals in Wuhan experienced COVID-19 outbreaks, marking the first time such units faced a nosocomial COVID-19 outbreak.^5,21^ Mental health practitioners, in Wuhan, encountered difficulties because of lacking guidelines, resources and insufficient training to provide mental health services during the COVID-19 pandemic.^21^

In-patient psychiatric outbreaks were reported to be because of nosocomial or community-acquired transmission.^8^ A study describing a COVID-19 outbreak in a psychiatric in-patient unit found that 41% of COVID-19 infections were because of nosocomial spread.^8^ Staff were carriers, so monitoring symptoms, hand hygiene, personal protective equipment and training were important.^2,8^ Early in the pandemic, researchers in California, US, found that psychiatric units managing patients with SMI experienced higher COVID-19 doubling times than general hospitals.^22^ Tailored outbreak management policies were required, which included strategies for prevention, early detection, proactive testing, patient education, social distancing and improving staff skills concerning infection-prevention policies.^2,4,8^

A review by Vai et al. investigated the correlation between mental disorders and the risk of COVID-19-related mortality, hospitalisation and admission to the intensive care unit (ICU).^6^ They found that individuals with SMI, particularly those with psychotic disorders, were at an increased risk of COVID-19 mortality. Additionally, the SMI population had a higher likelihood of hospitalisation, but not ICU admission, compared to the general population.^6^ Social and lifestyle factors, along with medical comorbidities, could exacerbate COVID-19 outcomes in this group of patients.^6^ This contrasts with the findings from a study in Istanbul, Turkey and another from Connecticut, US, where none of the patients had complications because of COVID-19, and all recovered.^13,20^

According to our knowledge, no published research exists exploring COVID-19 disease in in-patients in a psychiatric hospital in South Africa. This research project described the profile of patients with SMI and COVID-19 admitted to a specialist psychiatric hospital in South Africa from 01 April 2020 to 30 September 2021. The objectives were to describe the sample population’s demographic and psychiatric clinical characteristics and their COVID-19 symptoms, severity and need for transfer to a general medical hospital. This research is important as institutions caring for those with SMI require information regarding altered vulnerabilities and unique treatment needs of this population during the COVID-19 pandemic.

## Research methods and design

### Study design

This was a retrospective, descriptive, cross-sectional study in the form of a folder review. The study cohort included all the patients admitted to Vàlkenberg Hospital (VBH) from the acute and forensic services who tested positive for SARS-CoV-2 (severe acute respiratory syndrome coronavirus 2) from 01 April 2020 until 30 September 2021.

### Study setting

Vàlkenberg Hospital is a government-funded specialist psychiatric hospital. The 370-bed institution in Cape Town dates back to 1891 and comprises 145 forensic service beds and 225 acute service beds. It provides in-patient and out-patient services.

The acute services primarily deal with patients with SMI admitted under the *Mental Health Care Act* (MHCA) (Act 17 of 2002) who were aged 18–60. The forensic service admits patients for forensic observation and state patients as mandated in the *Criminal Procedure Act* Sections 77–79 (Act 51 of 1977). This service caters for patients from the age of 18 years and older.

Vàlkenberg Hospital limited non-elective admissions because of COVID-19. Admission criteria were stricter, and all patients were screened for symptoms. Asymptomatic patients were admitted with COVID-19 negative patients, while positive patients with mild symptoms were sent to VBH’s COVID-19 service.

All VBH in-patients were screened daily via daily review of vital signs and the VBH COVID-19 patient screening tool form (Appendix 1). Patients suspected of COVID-19 were isolated as persons under investigation, and a polymerase chain reaction (PCR) test was conducted.

The consultant in charge of the VBH COVID-19 service developed a screening form for patients diagnosed with COVID-19 (Appendix 2). The form was based on an existing screening tool reported in the literature.^23^ The process aimed to manage COVID-19 patients safely. Appendix 2 evaluated signs and scored patients. Mild symptoms (score 0–4) were treated in the VBH COVID-19 service. Severe symptoms (score ≥ 5 or 0–4 with comorbidities) were transferred to a medically equipped ward at Groote Schuur Hospital.

### Study population and sampling strategy

This is a non-probability convenience sample.

Inclusion criteria:

- The in-patients admitted to the acute and forensics services at VBH.
- Age 18 years and older.
- Sex: male and female.
- These patients must have had a positive SARS-COV-2 PCR test requiring isolation.

Exclusion criteria:

- Those who had a COVID-19 diagnosis before VBH admission but were not treated by the VBH COVID-19 service as they had completed their isolation period.

### Data collection

The completed COVID-19 forms (Appendix 2) and folders of the patients who required admission to VBH and tested positive for COVID-19 from 01 April 2020 to 30 September 2021 were reviewed.

The study focused on variables considered to affect the outcome of COVID-19, which resulted in more severe disease. Factors included:

- sociodemographic variables: age and sex.
- psychiatric variables: psychiatric diagnosis, current substance use: cigarettes, alcohol, cannabis, methamphetamine.
- medical variables: human immunodeficiency virus (HIV) status, body mass index (BMI), hypertension, diabetes mellitus, cardiovascular disease, chronic lung disease, previous tuberculosis (TB), COVID-19 symptoms during the quarantine period.
- outcomes: recovery from acute COVID-19 (during the quarantine period), transferred to a medical ward or demised.

### Data analysis

The data were extracted, captured and analysed using a statistical software package, Statistica 14.0. Descriptive statistics such as frequencies, median and mean were used to examine the distribution of demographics, psychiatric diagnosis, current substance use, medical comorbidities and COVID-19 symptoms.

Thereafter, associations between patient outcomes and several variables were calculated. The variables included:

- principal psychiatric diagnosis
- age and sex
- medical comorbidities
- substance use
- vital signs

Associations were run between the group transferred to the medical wards and those not transferred, comparing the following variables: sociodemographic, psychiatric and medical variables.

For categorical variables, chi-square and Fisher exact tests were used to determine significant associations. For continuous measurements, ANOVA and correlation analyses were conducted. The threshold for statistical significance was set at *p* = 0.05.

### Ethical considerations

This study was conducted in accordance with the Declaration of Helsinki (2013),^24^ the South African Good Clinical Practice Guidelines (DoH 2006)^25^ and The South African Department of Health Ethics in Health Research: Principles, Processes and Structures (DoH 2015).^26^

Ethics approval was obtained from the University of Cape Town Human Research Ethics Committee (HREC 771/2021). Approval was obtained from VBH and the Provincial Government of the Western Cape (WC_202112_015).

This was a retrospective dataset and folder review that involved no patient contact. As the data collected were anonymised, no informed consent was sought.

## Results

Over the study period, 254 participants tested positive for SARS-COV-2. Seventeen patients (7%) required transfer to the medical hospital. Two hundred and fifty-two (99%) patients recovered from acute COVID-19 during the isolation period, and 2 (1%) died.

**FIGURE 1:**
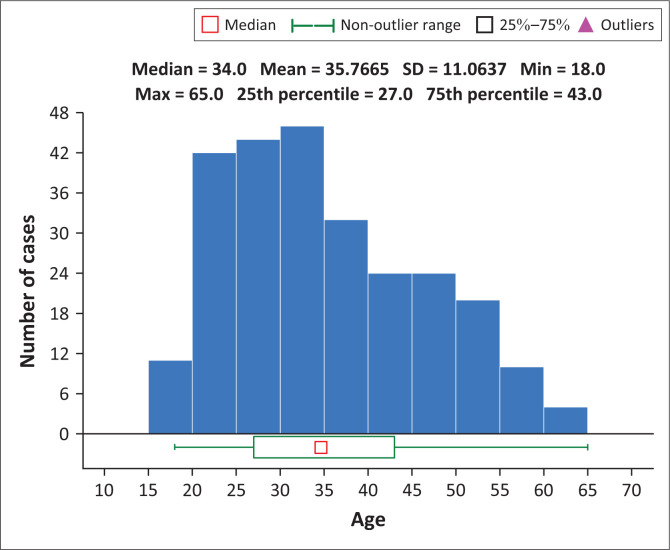
Age distribution of the study sample (*N* = 254).

The sample included 191 (75%) male and 63 (25%) female patients. The study population had a mean age of 35.7 (s.d. = 11.02), ranging from 18 to 65 years (Figure 1). The mean age of those transferred to the medical hospital was 42.06 (s.d. = 14.67) years, and those not transferred were 35.27 (s.d. = 10.61) years. Patients transferred to the medical hospital were significantly older (*p* = 0.01) (Table 1).

**TABLE 1:** Description of the demographic, psychiatric and medical profile of the sample (*N* = 254).

Variable	*n*	%	Mean	s.d.	Not transferred				Transferred				*p* = 0.05
					*n*	%	Mean	s.d.	*n*	%	Mean	s.d.	
Age	-	-	35.72	11.02	-	-	35.27	10.61	-	-	42.06	14.67	**0.01** (t)
**Sex**													
Male	191	75	-	-	180	75.9	-		11	64.7	-	-	0.38 (f)
Female	63	25	-	-	57	24.1	-		6	35.3	-	-	-
**Service**													
Acute	214	84	-	-	198	83.5	-		15	88.2	-	-	1.00 (f)
Forensics	41	16	-	-	39	16.5	-		2	11.8	-	-	-
**Psychiatric diagnosis**											-	-	
Schizophrenia	94	37	-	-	88	37.1	-	-	6	35.3	-	-	0.88 (c)
Bipolar disorder	54	21	-	-	48	20.3	-	-	6	35.3	-	-	0.21 (f)
SIPD/SIMD	41	16	-	-	39	16.5	-	-	2	11.8	-	-	1.00 (f)
Depression	7	3	-	-	7	3.0	-	-	0	0.0	-	-	1.00 (f)
Schizoaffective disorder	49	19	-	-	46	19.4	-	-	3	18.8	-	-	1.00 (f)
Other	59	23	-	-	56	23.6	-	-	3	17.6	-	-	0.77 (f)
**Comorbid substance use**													
Nicotine	181	72	-	-	169	71.6	-	-	12	70.6	-	-	1.00 (f)
Cannabis	151	59	-	-	140	59.3	-	-	10	58.8	-	-	0.97 (c)
Methamphetamine	92	36	-	-	87	36.9	-	-	5	29.4	-	-	0.54 (c)
Methaqualone	38	15	-	-	36	15.3	-	-	2	11.8	-	-	1.00 (f)
Alcohol	44	17	-	-	40	16.9	-	-	4	23.5	-	-	0.51 (f)
Other	26	10	-	-	26	11.1	-	-	0	0.0	-	-	0.23 (f)
**Comorbid medical disorders**													
Diabetes mellitus	10	4	-	-	10	4.2	-	-	0	0.0	-		1.00 (f)
Hypertension	28	11	-	-	25	10.6	-	-	3	17.6	-		0.41 (f)
Cardiovascular disease	4	2	-	-	3	1.3	-	-	1	5.9	-		0.24 (f)
Chronic lung disease	14	6	-	-	13	5.5	-	-	1	5.9	-		1.00 (f)
HIV	18	7	-	-	16	6.8	-	-	2	11.8	-		0.35 (f)
Previous PTB	15	6	-	-	12	5.1	-		3	17.6	-		0.07 (f)
BMI > 30	10	4	-	-	9	3.8	-	-	1	5.9	-	-	0.51 (f)
Other	51	20	-	-	44	18.6	-	-	7	41.2	-	-	**0.05** (f)

BMI, body mass index; s.d., standard deviation; HIV, human immunodeficiency virus; SIPD, substance induced psychotic disorder; SIMD; substance induced mood disorder; PTB, pulmonary tuberculosis.

(t): Independent sample *T*-test. (f): Fisher’s exact test. (c): chi square test.

The study population consisted of 214 (84%) patients from the acute service and 41 (16%) patients from the forensic service at VBH. Of those patients who required transfer, 15 (88.2%) were from the acute service and 2 (11.8%) from the forensic service.

Ninety-four (37%) patients had a diagnosis of schizophrenia, 59 (23%) were grouped as other (which includes anxiety disorders, personality disorders and psychosis secondary to another medical condition), 54 (21%) had bipolar disorder and 49 (19%) had schizoaffective disorder. Both principal and secondary diagnosis were factored in (Table 1).

The most common medical comorbidity was hypertension in 28 (11%) patients. The only comorbidity to have statistical significance associated with transfer to the medical hospital was ‘other’ (*p* = 0.05) (Table 1). The comorbidities grouped as ‘other’ included neurological conditions, thyroid disorders, autoimmune disorders and dermatological disorders. At the time of the study, none of the comorbidities in the ‘other’ group were associated with COVID-19 severity.

The most common substance used was nicotine by 181 (72%) patients, followed by cannabis in 151 (59%) and methamphetamine in 92 (36%).

One hundred and fifty-six (62%) patients were symptomatic for COVID-19 (Table 2).

**TABLE 2:** Description of coronavirus disease 2019 symptoms (*N* = 252).

Symptom	*n*	%	Not transferred		Transferred		*p* = 0.05
			*n*	%	*n*	%	
Asymptomatic	96	38	94	40.0	2	11.8	0.02 (c)
**Symptomatic**	156	62	141	60.0	15	88.2	-
Cough	74	29	68	28.9	6	35.3	0.59 (f)
Sore throat	69	27	64	27.2	5	29.4	0.79 (f)
Myalgia	65	26	58	24.7	7	41.2	0.15 (f)
Fever	51	20	46	19.7	5	29.4	0.35 (f)
Shortness of breath	22	9	17	7.3	5	29.4	**0.01** (f)
Diarrhoea	17	7	16	6.8	1	5.9	1.00 (f)
Anosmia	10	4	10	4.3	0	0.0	1.00 (f)
Other	72	29	66	28.1	6	35.3	0.58 (f)

Table 3 displays a description of the patients’ vital signs. The majority of patients were alert (*n* = 241; 96%). Patients who were not transferred were significantly more likely to be alert (*p* = 0.002). Most patients had a normal respiratory rate between 12 and 20 breaths per minute (*n* = 229; 92%). Patients who were not transferred were significantly more likely to have a normal respiratory rate (*p* < 0.001). On the other hand, patients who were transferred were significantly more likely to have a respiratory rate above 20 (*p* < 0.001).

**TABLE 3:** Description of the vital signs (*N* = 253).

Vital sign	*n*	%	Not transferred		Transferred yes		*p* = 0.05
			*n*	%	*n*	%	
**Alert**							**0.002**
Yes	241	96	228	97.9	13	76.5	-
No	9	4	5	2.1	4	23.5	-
**Respiratory rate**							**< 0.010**
< 12	0	0	0	0.0	0	0.0	-
12–20	229	92	221	94.8	8	47.1	**< 0.001**
20–24	18	7	12	5.2	6	35.3	**< 0.001**
> 25	3	1	0	0.0	3	17.6	**< 0.001**
**Oxygen saturation**							**< 0.001**
≤ 93%	11	4	5	2.1	6	35.3	-
≥ 94%	237	95	226	97.0	11	64.7	-
**Oxygen needed**							**< 0.001**
Yes	10	4	2	0.9	8	47.1	-
No	240	96	231	99.1	9	52.9	-
**Systolic blood pressure**							
< 100	14	6	12	5.2	2	11.8	0.250
100–140	223	89	210	90.1	13	76.5	0.180
> 140	12	5	10	4.3	2	11.8	0.190
**Heart rate**							
< 60	0	0	1	0.4	0	0.0	1.000
60–100	191	76	184	79.0	7	41.2	**0.001**
> 101	57	23	47	20.2	10	58.8	**< 0.001**
**Temperature**							
< 35.9	17	7	16	6.9	1	5.9	1.000
36–37.4	190	76	179	76.8	11	64.7	0.250
> 37.5	43	17	38	16.3	5	29.4	0.180

Eleven (4%) patients had low oxygen saturation below 94%, and 10 (4%) required supplemental oxygen. Low oxygen and requiring supplemental oxygen were significantly associated with transfer to the medical hospital (*p* < 0.001) (Table 3).

Most patients had temperatures in the normal range of 36–37.5 degrees Celsius (*n* = 193; 76%).

The majority of the patients had normal heart rates between 60 and 100 beats per minute (*n* = 191; 76%). Patients transferred to a medical hospital were significantly more likely to have a heart rate of 60–100 compared to patients not transferred (*p* = 0.001). However, only 57 (23%) patients had increased heart rates above 100 beats per minute. Patients who were transferred were more likely to have an increased heart rate (*p* < 0.001) (Table 3).

Two hundred and twenty-three (89%) patients had systolic blood pressures within the normal range of 100–139 millimetres of mercury, and only 12 patients (5%) had a high systolic blood pressure above 140 millimetres of mercury. From the vital signs described in Table 3, the following significant associations are reported: Patients who were not transferred (remained at VBH) were significantly more likely to (1) be alert (*p* = 0.002), (2) have a normal respiratory rate (*p* < 0.001), (3) not need oxygen (*p* < 0.001) and (4) have a normal heart rate (*p* = 0.001).

## Discussion

In this study, we described the psychiatric, medical profile and clinical outcomes of patients with SMI and COVID-19 admitted to a specialist psychiatric hospital in South Africa. Most patients were male and in their thirties, receiving care in the acute service. Schizophrenia and bipolar disorder were the prevailing diagnoses, and nicotine was the most frequently used substance. Despite this population’s heightened risk of COVID-19 complications, only a few were transferred to other facilities, and only two individuals succumbed to the illness. These findings are remarkable, particularly considering prior reports indicating worse outcomes for those with SMI and COVID-19.^6,7,27,28,29^

Throughout the study period, it was observed that a higher number of male patients received a COVID-19 diagnosis in comparison to female patients. This variance in figures could be attributed to the fact that VBH allocates more beds to male patients in the acute service. More patients were from the acute service. The acute service had more COVID-19 cases because of the higher frequency of patient admissions, resulting in increased vulnerability to infections and more difficulty in controlling outbreaks. This finding could also suggest that the hospital’s forensic unit policy of limiting admissions during the pandemic was effective. Furthermore, the absence of female forensic state patients at the hospital contributed to more male patients. The prevalence of male patients is consistent with the patient profiles of other specialised psychiatric hospitals in South Africa.^30,31^ Research has suggested that being male may be a risk factor for susceptibility to infection and severe illness resulting from COVID-19.^32^ Notably, only a small percentage of the total cohort was transferred to a medical hospital, and there was no significant difference in transfer rates between males and females.

The cohort had an average age of 35.7 years and a median age of 34. This finding is not surprising given that it is common for acute psychiatric services to admit younger patients and that the hospital forensic unit adopted a policy of limiting admissions during the pandemic.^30,31^

Our study revealed that patients transferred to a medical hospital were significantly older than those that recovered at VBH. This aligns with previous research indicating that older individuals experience more severe illness and worse outcomes.^33,34,35^

In this study, schizophrenia, bipolar disorder and schizoaffective disorder were the most common psychiatric diagnoses. This is similar to other SMI cohorts admitted to psychiatric hospitals in South Africa.^31^ Evidence suggests that those with SMI have a higher mortality rate and reduced life expectancy than the general population.^29^ South Africa’s general population experienced high fatality because of COVID-19.^36^ Thus, we expected this population to be more vulnerable to COVID-19 and have poorer outcomes.^6,29^ The study found no significant link between psychiatric diagnoses and transfer to a medical hospital for COVID-19. However, it is possible that the results were influenced by the young age of the study group and the low occurrence of medical comorbidities. A few studies outside South Africa have raised concerns about the severity of COVID-19 in the SMI population, but this study showed that most infected patients were able to be treated at VBH and did not need to be transferred to a medical hospital.^6,28,29^ The majority of the population had mild COVID-19 and experienced positive medical outcomes, with few fatalities. Similar findings have been reported in other psychiatric in-patient settings.^13,20^ The similarities in outcomes may be because of comparable age, disease severity and comorbidity profiles.^20^

Our sample revealed that a significant number of individuals presented with symptoms that are commonly associated with COVID-19.^37^ Interestingly, only a small subset of patients displayed anosmia, initially believed to be a hallmark symptom of SARS-COV-2.^38^ It is worth noting that most symptomatic patients reported experiencing a range of symptoms that were classified as ‘other’. These findings highlight the difficulties the SMI population may encounter in comprehending and articulating physical symptoms.^8^ Most symptoms were elicited through history taking and assessment of basic vital signs. This emphasises the importance of medical history taking and physical examination as a continuous process throughout the contact with psychiatric services.^39^ The only COVID-19 symptom that had statistical significance with needing to be transferred to a medical facility was shortness of breath. This is in keeping with respiratory distress and indicates a more severe COVID-19.^34^

The vital signs significantly associated with being transferred to a medical facility were heart rate, low oxygen saturation, needing oxygen, respiratory rate and alertness. These findings are associated with more severe COVID-19 and a greater need for management at a medically equipped facility.^23^ The group that required transfer was more likely to have abnormal vital signs, which is expected. However, most of the study population had vitals within normal limits. This suggests that most participants had a mild form of COVID-19, and the COVID-19 screening form (Appendix 2) appropriately identified those who needed to be transferred to a medical hospital early and those who could be treated at VBH.

Our study population reported nicotine as the most common substance used. High rates of nicotine use are prevalent in psychiatry inpatient settings in South Africa.^40,41^ This is especially seen in the male population.^40^ Smoking is a leading cause of preventable death, with a higher prevalence of conditions such as malignancy, cardiovascular disease and chronic obstructive pulmonary disease, increasing morbidity and mortality.^41^ During the COVID-19 pandemic, smoking cigarettes was initially considered a significant risk factor, prompting the South African government to ban cigarette sales early in the pandemic.^42,43^ This was implemented with concerns of significant respiratory compromise in nicotine smokers infected with COVID-19 and increasing the risk of virus transmission. Shortness of breath, low oxygen saturation and oxygen requirement were significantly associated with transfer, indicating more severe disease requiring medical care. Smoking was associated, in theory, with severe disease.^42^ However, in our sample, we did not find that association as only a few patients required transfer to a medical hospital.

A significant prevalence of cannabis and methamphetamine consumption was exhibited in the study cohort. It has been observed that individuals who have SMI tend to use multiple substances.^30,44^ It should be noted that substance use can have detrimental effects on the respiratory, cardiovascular and immune systems and can heighten the likelihood of severe COVID-19 and unfavourable outcomes.^45,46^ Nonetheless, only a minority of the participants experienced severe COVID-19, and thus, just a few needed to be relocated to an alternative facility.

The SMI population is at high risk for medical comorbidities because of their poor lifestyle factors and the side effects of psychopharmacological agents.^27,47^ It is worth noting that, apart from the category ‘other’, the study population had a low prevalence of medical comorbidities. Our sample’s lower rates of medical comorbidities may be attributed to the younger age group, which could also explain the lack of severe COVID-19 cases and the positive outcomes observed in our study. The category ‘other’ showed statistical association with the need to be transferred to a medical hospital, and previous pulmonary tuberculosis (PTB) showed a trend towards significance, suggesting that those who were transferred were more likely to have had previous TB. The literature has suggested a correlation between multimorbidity and severe COVID-19, as well as a poorer prognosis.^29,33^

In our study, most of our patients fared well, except for two individuals who passed away. These two patients were from the long stay ward, over 40 years old and with multiple underlying medical conditions. This is consistent with findings that older age and multiple medical comorbidities are associated with severe COVID-19.^29^

When analysing the results of this cross-sectional study, it is essential to consider certain limitations. Specifically, the study examined the link between COVID-19 exposure and outcome simultaneously, making interpreting the associations challenging. Additionally, the accuracy of the data collected relied on the form completed by the treating clinician at the time of admission to the VBH COVID-19 service. Other limitations include the study being conducted at a single site with a relatively small population size. Only medically screened patients who were physically stable were accepted for transfer into VBH, which could introduce bias. Finally, the study was conducted in a specific population within an urban hospital with specific geographic drainage, so the findings may not represent the larger population.

Our study highlighted the importance of comprehensive history taking, physical examination and the use of screening tools in managing COVID-19 patients at VBH during the pandemic.

## Conclusion

This study examined the outcomes of COVID-19 in patients with SMI who were admitted to a specialist psychiatric hospital. Despite concerns, most patients had mild illnesses and recovered. More research is needed to understand how psychiatric units are vulnerable to infectious diseases and to minimise their spread.

## Appendix 2

**TABLE 1-A2:** Valkenberg Hospital COVID19 TRACKER 2020

**Patient sticker if available**								
Demographics	Name:	-	-	-	-	-	-	-
	Folder Number:	-	-	-	-	-	-	-
	MaleFemale [M /F]	-	-	-	-	-	-	-
	DateOfBirth: [DDMMYYYY]	-	-	-	-	-	-	-
MHCA	Referral Hospital:	-	-	-	-	-	-	-
	DateForm06: [DDMMYYYY]	-	-	-	-	-	-	-
	Admission date to VBH: [DDMMYYYY]	-	-	-	-	-	-	-
	Date of referral to general hospital [DDMMYYY]	-	-	-	-	-	-	-
Psychiatric diagnoses		-	-	-	-	-	-	-
**Comorbid substance use disorders**								
COVID19	DateOfSymptomOnset:[DDMMYYYY]	-	-	-	-	-	-	-
		Yes	No					
	Asymptomatic	-	-	-	-	-	-	-
Symptoms	PresentingSymptomType1: Cough	-	-	-	-	-	-	-
	PresentingSymptomType2: Sore Throat	-	-	-	-	-	-	-
	PresentingSymptomType3: Shortness of breath	-	-	-	-	-	-	-
	PresentingSymptomType4: Anosmia	-	-	-	-	-	-	-
	PresentingSymptomType5: Fever	-	-	-	-	-	-	-
	PresentingSymptomType6: Myalgia	-	-	-	-	-	-	-
	PresentingSymptomType7: Diarrhoea	-	-	-	-	-	-	-
	PresentingSymptomType8: Other	-	-	-	-	-	-	-
	Quarantined ward	-	-	-	-	-	-	-
	DateOfTestTaken: [DDMMYYYY]	-	-	-	-	-	-	-
	DateTestResultPositive: [DDMMYYYY]	-	-	-	-	-	-	-
		Yes	No					
Comorbidity	Diabetes Mellitus	-	-	-	-	-	-	-
	Hypertension	-	-	-	-	-	-	-
	Cardiovascular disease	-	-	-	-	-	-	-
	Chronic lung disease: specify	-	-	-	-	-	-	-
	HIV	-	-	-	-	-	-	-
	Smoker	-	-	-	-	-	-	-
	Previous TB (specify year if known)	-	-	-	-	-	-	-
	BMI	-	-	-	-	-	-	-
Other comorbidity		-	-	-	-	-	-	-
**Transfer to general hospital**								
Physical symptoms		3	2	1	0	1	2	3
	Age	-	-	-	< 65	-	-	> 65
	Respiratory rate (/min)	< 9	-	9–11	12-20	-	20-24	> 25
	Oxygen sats on room air (%)	< 92	92-93	94–95	> 95	-	-	-
	Oxygen supply necessary	-	yes		no	-	-	-
	Systolic blood pressure mmHg	< 91	91–100	101–110	111–219	-	-	> 219
	Pulse (/min)	-	91–100	101–110	111–219	-	-	> 219
	Level of awareness	-	-		alert	-	-	altered
	Temperature (⁰C)	< 35	-	35–36	36–38	38–39	> 39	-
	Total	-	-	-	-	-		-
	0-4 Remain at VBH with bi-daily vitals and saturation monitoring	-	-	-	-	-		-
	0-4 with co-morbidities: Discuss with consultant	-	-	-	-	-		-
	5 or more: Transfer to designated medical facility	-	-	-	-	-		-
		-	-	-	-	-	Yes	No
Psychiatric	Responding to psychotic symptoms	-	-	-	-	-	-	-
Symptoms	Disinhibited	-	-	-	-	-	-	-
	Aggression-verbal	-	-	-	-	-	-	-
	Aggression-physical	-	-	-	-	-	-	-
	Disorganised	-	-	-	-	-	-	-
	Ongoing self-harm	-	-	-	-	-	-	-
	Suicidal intent	-	-	-	-	-	-	-
	Ongoing suicidal ideation	-	-	-	-	-	-	-
	Agitated/restless	-	-	-	-	-	-	-
Medication	Requires regular sedation	-	-	-	-	-	-	-
	Special motivation (e.g. Consta)	-	-	-	-	-	-	-
	Recent accuphase [DDMMYYYY]	-	-	-	-	-	-	-
	Last depot [DDMMYYYY]	-	-	-	-	-	-	-
	Name, dose and frequency	-	-	-	-	-	-	-
